# Supplementary material for: Translation, Adaptation, and Validation of the Modified Thai Version of Champion’s Health Belief Model Scale (MT-CHBMS)
Source: Healthcare (Basel). 2022 Dec 31;11(1):128. doi: 10.3390/healthcare11010128 (PMC9819080; doi:10.3390/healthcare11010128)
Supplement: Supplementary file 1 [file healthcare-11-00128-s001.zip › Supplement1.pdf]

Table S1. Univariate sample statistics of the Modified Thai version of CHBMS items										
		N	Minimum	Maximum	Mean	SD	Skewness		Kurtosis	
		Statistic	Statistic	Statistic	Statistic	Statistic	Statistic	SE	Statistic	SE
1	S1	130	1	5	2.50	1.122	.150	.212	-.875	.422
2	S2	130	1	5	2.41	1.098	.346	.212	-.678	.422
3	S3	130	1	5	2.57	1.092	.092	.212	-.785	.422
4	S4	130	1	5	2.44	1.107	.350	.212	-.612	.422
5	S5	130	1	5	2.38	1.073	.345	.212	-.546	.422
6	se1	130	1	5	3.58	1.105	-.795	.212	.208	.422
7	se2	130	1	5	3.14	1.002	-.283	.212	-.033	.422
8	se3	130	1	5	3.28	1.057	-.577	.212	-.161	.422
9	se4	130	1	5	3.15	1.089	-.275	.212	-.540	.422
10	se5	130	1	5	2.74	1.111	.053	.212	-.809	.422
11	se6	130	1	5	3.42	1.187	-.534	.212	-.592	.422
12	se7	130	1	5	2.72	1.214	.364	.212	-.828	.422
13	beb1	130	1	5	4.02	.747	-1.159	.212	3.287	.422
14	beb2	130	1	5	3.98	.871	-1.469	.212	3.438	.422
15	be3	130	1	5	4.00	.872	-1.355	.212	2.529	.422
16	be4	130	1	5	4.15	.818	-1.487	.212	3.804	.422
17	be5	130	1	5	4.08	.813	-1.374	.212	3.474	.422
18	be6	130	1	5	4.10	.746	-1.304	.212	3.873	.422
19	bm1	130	3	5	4.25	.626	-.246	.212	-.609	.422
20	bm2	130	2	5	4.25	.697	-.671	.212	.358	.422
21	bm3	130	2	5	4.29	.731	-.760	.212	.111	.422
22	bm4	130	1	5	4.23	.793	-1.007	.212	1.381	.422
23	bm5	130	1	5	4.12	.826	-.904	.212	1.007	.422
24	bm6	130	2	5	4.21	.775	-.888	.212	.680	.422
25	barb1	130	1	5	4.15	.992	-.993	.212	.315	.422
26	barb2	130	1	5	3.49	1.080	-.261	.212	-.554	.422
27	barb3	130	1	5	4.20	.952	-1.125	.212	.854	.422
28	barb4	130	1	5	3.99	1.060	-.777	.212	-.465	.422
29	barb5	130	1	5	3.78	1.064	-.500	.212	-.653	.422
30	barb6	130	1	5	4.01	1.045	-.925	.212	.199	.422
31	barm1	130	1	5	3.75	1.072	-.529	.212	-.507	.422
32	barm2	130	1	5	4.25	.890	-1.245	.212	1.620	.422
33	barm3	130	1	5	3.94	1.002	-.814	.212	.260	.422
34	barm4	130	1	5	3.68	1.086	-.302	.212	-.902	.422
35	barm5	130	1	5	3.45	1.195	-.286	.212	-.920	.422
36	beu1	130	1	5	4.20	.687	-.864	.212	2.472	.422
37	beu2	130	1	5	4.12	.784	-1.089	.212	2.551	.422
38	beu3	130	1	5	4.18	.734	-1.021	.212	2.333	.422

39	beu4	130	1	5	4.10	.888	-1.346	.212	2.740	.422
40	beu5	130	1	5	3.98	.976	-1.172	.212	1.560	.422
41	beu6	130	1	5	4.04	.893	-1.072	.212	1.340	.422
42	bau1	130	1	5	3.58	1.105	-.480	.212	-.414	.422
43	bau2	130	1	5	4.10	.905	-.837	.212	.270	.422
44	bau3	130	1	5	3.98	.923	-.689	.212	-.014	.422
45	bau4	130	2	5	3.93	.950	-.467	.212	-.760	.422
46	bau5	130	1	5	3.44	1.175	-.273	.212	-.942	.422
47	I1	130	1	5	3.89	.838	-1.402	.212	3.144	.422
48	I2	130	1	5	3.75	.836	-.883	.212	.727	.422
49	I3	130	1	5	3.71	.867	-1.062	.212	.941	.422
50	I4	130	1	5	3.70	.903	-1.033	.212	.622	.422
51	I5	130	1	5	3.55	1.050	-.819	.212	-.057	.422
52	I6	130	1	5	3.40	1.132	-.774	.212	-.277	.422
53	I7	130	1	5	2.78	1.161	-.022	.212	-1.162	.422
54	I8	130	1	5	3.46	.966	-.676	.212	-.166	.422
55	I9	130	1	5	3.15	1.191	-.359	.212	-.974	.422
56	I10	130	1	5	3.46	1.094	-.857	.212	-.064	.422
57	I11	130	1	5	3.48	1.028	-.849	.212	.014	.422
58	M1	130	2	5	4.42	.644	-.826	.212	.476	.422
59	M2	130	3	5	4.58	.582	-1.023	.212	.071	.422
60	M3	130	3	5	4.42	.608	-.540	.212	-.599	.422
61	M4	130	3	5	4.50	.560	-.537	.212	-.754	.422
62	M5	130	2	5	4.28	.685	-.580	.212	-.127	.422
63	M6	130	1	5	3.91	.968	-.958	.212	.730	.422
64	M7	130	1	5	4.34	.763	-1.405	.212	2.967	.422
