# Supplementary material for: Translation, Adaptation, and Validation of the Modified Thai Version of Champion’s Health Belief Model Scale (MT-CHBMS)
Source: Healthcare (Basel). 2022 Dec 31;11(1):128. doi: 10.3390/healthcare11010128 (PMC9819080; doi:10.3390/healthcare11010128)
Supplement: Supplementary file 1 [file healthcare-11-00128-s001.zip › Supplement2.pdf]

Table S2. Error terms correlation

No.	Pair of error terms	T-CHBMS				MT- CHBMS				
		Estimate	S.E.	Est./S.E.	P-Value		Estimate	S.E.	Est./S.E.	P-Value
1	S2-S1	0.506	0.052	9.688	<.001		0.496	0.053	9.38	<.001
2	S4-S3	0.545	0.073	7.476	<.001		0.546	0.074	7.355	<.001
3	SE3-SE2	0.518	0.066	7.855	<.001		0.502	0.07	7.182	<.001
4	SE7-SE6	0.421	0.073	5.744	<.001		0.417	0.074	5.661	<.001
5	BEB2-BEB1	0.614	0.05	12.286	<.001		0.631	0.051	12.437	<.001
6	BM2-BM1	0.741	0.107	6.92	<.001		0.711	0.119	5.953	<.001
7	BM5-BM4	0.385	0.068	5.63	<.001	BM5-BM3	-1.781	0.702	-2.537	0.011
8						BM4-BM1	-0.31	0.141	-2.198	0.028
9	BARB2-BARB1	0.419	0.087	4.798	<.001		0.401	0.085	4.697	<.001
10	BARB2-BARB4	-0.412	0.169	-2.434	0.015		-0.495	0.183	-2.704	0.007
11	BARB3-BARB1	0.575	0.058	9.963	<.001		0.579	0.061	9.464	<.001
12	BARM4-BARM3	0.44	0.083	5.276	<.001		0.427	0.074	5.771	<.001
13	I2-I1	0.539	0.066	8.226	<.001		0.526	0.07	7.491	<.001
14	I2-I9	-0.357	0.083	-4.321	<.001		-0.299	0.085	-3.531	<.001
15	I4-I3	0.711	0.114	6.226	<.001		0.631	0.051	12.437	<.001
16	I6-I5	0.858	0.023	38.118	<.001		0.861	0.023	36.908	<.001
17	I6-I7	0.531	0.052	10.248	<.001		0.555	0.053	10.532	<.001
18	I9-I1	-0.538	0.106	-5.083	<.001		-0.52	0.114	-4.549	<.001
19	I9-I8	0.457	0.079	5.797	<.001		0.479	0.076	6.267	<.001
20	I9-I11	0.648	0.069	9.381	<.001		0.671	0.067	9.994	<.001
21	I11-I8	0.548	0.066	8.284	<.001		0.561	0.065	8.643	<.001

T-CHBMS = Thai version of the champion's health belief model scale, TM-CHBMS = modified Thai version of the champion's health belief model scale
